# SMS-based smoking cessation intervention among university students: study protocol for a randomised controlled trial (NEXit trial)

**DOI:** 10.1186/s13063-015-0640-2

**Published:** 2015-04-08

**Authors:** Ulrika Müssener, Marcus Bendtsen, Nadine Karlsson, Ian R White, Jim McCambridge, Preben Bendtsen

**Affiliations:** Department of Medicine and Health Sciences, Linköping University, 581 83 Linköping, Sweden; Department of Computer and Information Science, Linköping University, 581 83 Linköping, Sweden; MRC Biostatistics Unit, Cambridge Institute of Public Health, Robinson Way, Cambridge, CB2 0SR UK; Department of Health Sciences, University of York, Heslington, YO10 5DD UK; Department of Medical Specialist and Department of Medicine and Health Sciences, Linköping University, Motala, 581 83 Linköping, Sweden

**Keywords:** Tobacco, Smoking cessation, Students, Text messages, Mobile phones, SMS

## Abstract

**Background:**

Most smoking efforts targeting young people have so far been focused on prevention of initiation, whereas smoking cessation interventions have largely been targeted towards adult populations. Thus, there is limited evidence for effective smoking cessation interventions in young people, even though many young people want to quit smoking. Mobile communication technology has the potential to reach large numbers of young people and recent text-based smoking cessation interventions using phones have shown promising results.

**Methods/design:**

The study aims to evaluate a newly developed text-based smoking cessation intervention for students in colleges and universities in Sweden. The design is a randomised controlled trial (RCT) with a delayed/waiting list intervention control condition. The trial will be performed simultaneously in all colleges and universities served by 25 student health care centres in Sweden. Outcomes will be evaluated after 4 months, with 2 cessation primary outcomes and 4 secondary outcomes. After outcome evaluation the control group will be given access to the intervention.

**Discussion:**

The study will examine the effectiveness of a stand-alone SMS text-based intervention. The intervention starts with a motivational phase in which the participants are given an opportunity to set a quit date within 4 weeks of randomisation. This first phase and the subsequent core intervention phase of 12 weeks are totally automated in order to easily integrate the intervention into the daily routines of student and other health care settings.

As well as providing data for the effectiveness of the intervention, the study will also provide data for methodological analyses addressing a number issues commonly challenging in Internet-based RCTs. For example, an extensive follow-up strategy will be used in order to evaluate the use of repeated attempts in the analysis, and in particular to explore the validity of a possible missing not at random assumption that the odds ratio between the primary outcome and response is the same at every attempt.

**Trial registration:**

ISRCTN: ISRCTN75766527, dated assigned 4 November 2014. Protocol version: Version 1, and date 7 November 2014.

**Electronic supplementary material:**

The online version of this article (doi:10.1186/s13063-015-0640-2) contains supplementary material, which is available to authorized users.

## Background

Worldwide, nearly 100.000 young people start smoking every day and in Sweden the annual number of new younger smokers is between 16,000 and 20,000 [1,2]. Longer-term reductions in the prevalence of smoking in the general population in Sweden have started to level off and in 2013 the proportions of daily smokers among both men and women were 11% [3]. In young people between 16 and 29 years of age the prevalence of daily smoking in 2013 was 12% among women and 7% among men, whereas occasional smoking was approximately twice as common in women and 3 times as common among men [3].

Smoking is responsible for more than 60 diseases and is globally the most important preventable cause of ill health and death. For every death related to smoking, more than 20 additional individuals will suffer from at least 1 serious smoking-related illness [4]. Tobacco is responsible for approximately 9.6% of the total disease burden in Sweden [5] and around 6,000 people die every year in Sweden due to smoking [1]. Many of the negative health effects of smoking develop after many years of smoking and there is a linear dose–response relationship between smoking and tobacco-induced diseases, such that the longer a person smokes the more likely the person will develop a smoking-related illness [1]. Most smokers start in their teens and over the course of a year most young smokers want to quit or cut down [6]. Among all smokers, around 65% in some studies want to quit and around half of all smokers make at least 1 quit attempt each year, but only 10% seek or gain access to evidence- based helping resources [7]. The addictive nature of smoking makes cessation difficult and the cost, time commitments and logistics associated with treatment are additional barriers [8].

Identifying effective interventions to help young people to quit smoking would have a major impact on population health. However, there is a limited amount of evidence for effective smoking cessation intervention in young people [2,9,10]. In a recent Cochrane review, 28 good quality studies were identified with about 6,000 young people included in various forms of tobacco cessation programmes. These include a variety of promising approaches, such as behavioural change support and motivational enhancement, but still the number of trials and participants are not large enough to judge which approaches are best implemented on a large scale [9]. Existing smoking cessation programmes also do not reach sufficient proportions of young smokers [9]. Thus, there is both a lack of knowledge on how best to reach young people and on how to effectively intervene with them, in order to reduce the number of young people still smoking [11].

During the last decade the development and dissemination of computerised health behaviour interventions have expanded exponentially, moving health behaviour change interventions from delivery in the health care sector into people’s homes and wherever they happen to be. An increasing number of Internet-based health behaviour change interventions show promising evidence of effectiveness in several life style areas [12-14].

In contrast to Internet-based interventions delivered on computers, mobile phone interventions have the capacity to interact in a more dynamic way with the individual with much greater frequency using short message service (SMS), more commonly known as text messages. Thus, mobile phone technology could provide a new mode of delivering personalised smoking cessation support. Since nearly all young people have a mobile phone and often use SMS to communicate with each other, SMS-based interventions could potentially be a vehicle for a large public health impact, even if only modestly effective [15-18].

At present, more than 50 smoking-cessation apps for smart phones are available to download. However, very few have evidence-based content [19]. Also, most apps need to be actively opened by the person. So, it is important not only to download the app but also to use it as intended [19]. This contrasts with SMS-based interventions where the person automatically receives a message with information or another intervention component and does not have to actively decide to continue accessing the intervention. This increases the potential for high levels of exposure and strong adherence to intervention content in real time in everyday settings.

Research on mobile phone-based interventions for smoking cessation has so far been very scarce. One of the first studies of a solely SMS-based intervention was performed in New Zealand in 2005 - the ‘Do u smoke after txt?’ study. The study showed a significant 6-week increased quit rate in the SMS group compared to the control group (28% versus 13%), whereas at 6 months the results were difficult to interpret due to missing values [18]. A later study, the ‘txt2Stop’ study from the UK, was based upon a modified version of the New Zealand intervention. Its pilot study showed at 6 months follow-up a self-reported quit rate of 26% in the intervention group compared with 12% in the control group [17], and in the main study the quit rate was 19.8% in the SMS intervention group compared to 13.5% in the control group. The biochemically verified abstinence rate was 10.7% in the SMS group and 4.9% in the control group [15].

In a recent Cochrane review only two additional studies besides the three studies above were identified; one of these also included the use of the Internet and other video messages sent via the mobile phone [20]. Since this review, five more randomised controlled trials (RCTs) have been published of solely SMS-based smoking cessation interventions [21-25]. Four of these showed promising results [21,22,24,25] although three were pilot studies not powered to detect significant differences between intervention and control group [22,24,25]. In the fifth study no significant differences were seen in short-term abstinence (assessed 8 weeks after randomisation) between a control group and participants receiving a 12-week SMS intervention. However, longer-term prolonged abstinence (assessed 6 months after randomisation) was significantly higher in the SMS group than in the control group (15.1% versus 8.9%) [23]. The central component of these interventions was daily text messages based upon various behaviour change theories and/or existing evidence-based guidelines for smoking cessation, during an intervention period of between 6 and 12 weeks.

In summary, although existing data are promising, proven effective smoking cessation interventions targeting young people are scarce and further research is needed to evaluate the effectiveness of innovative interventions capable of reaching young people [26,27]. Our aim is to present the study protocol of a RCT testing the effectiveness of a new SMS-based smoking cessation intervention for college and university students in Sweden.

## Methods/design

### Design

This is a 2-arm RCT study in which participants are randomised to an intervention (group 1) or a waiting list (group 2). In Additional file 1, the recommended items included in the reporting of the RCT are listed. After the 12-week intervention all participants in both groups will be asked to complete a follow-up questionnaire after which the control group will be given access to the intervention. The study will be performed simultaneously at all universities and colleges in Sweden.

### Objectives and hypotheses

The objective of the study is to evaluate the effectiveness of a SMS-based smoking cessation intervention. Participants in both the intervention and control group are told that they are free to seek any other treatment they want during the trial in the same manner as in previous studies [15-18].

Six hypotheses in total are being tested. The 2 primary hypotheses are that a greater proportion of participants in the intervention group than in the control group will report: 1) prolonged abstinence (8 weeks) and 2) recent abstinence (4 weeks) at follow-up. Secondary hypotheses are that the intervention group compared with the control group will report: 3) greater proportions of current abstinence (1 week); 4) a higher number of quit attempts; 5) greater use of other smoking cessation services; and 6) smoking fewer cigarettes among those still smoking at follow-up.

### Participants, setting and recruitment

**Figure 1:**
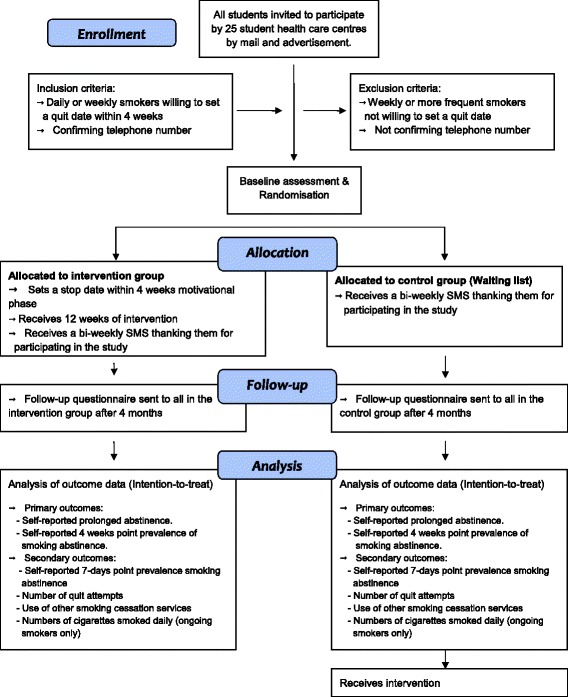
**Consolidated Standards of Reporting Trials (CONSORT) flow chart.** CONSORT flow chart illustrating all steps in the study from enrolment to allocation and follows up. Inclusion and exclusion criteria are also specified as well as outcome measures.

All 25 student health care centres responsible for preventive services for students in Sweden will participate in recruiting interested smokers during a 4-week period in October 2014, through advertisement at strategic places in each college and university. This includes leaflets, posters and information on the local website and in some cases the official Facebook account. Participants register their interest in taking part in the study by sending a SMS to a dedicated telephone number. Interested students will receive mail with more information about the study and how to participate. Also, 19 student health care centres will send an invitation by Email to all students at the end of October 2014 with 2 reminders with 1 week apart, allowing the participants to respond up to 7 days after the last reminder. This means that recruitment of participants will be finalised at the end of the second week of November 2014. A flowchart of the study recruitment procedure is given in Figure 1. Eligible participants are students who are daily or weekly smokers and willing to set a quit date for smoking cessation within the next 4-week period. Exclusion criteria are non-smokers/occasional smokers not smoking every week, and smokers not willing to set a quit date within 4 weeks.

### Randomisation and other study procedures

Smokers who respond to the invitation Email and want more information about the study will be referred to a page with more information about the study emphasising that only students who are daily or weekly smokers willing to set a stop date within a month are eligible for the study. Most previous SMS-based smoking cessation studies have used the inclusion criteria - willing to set a stop date with 2 to 4 weeks - facilitating recruitment of a more engaged population both in the intervention and control group and thereby avoiding a larger drop-out of randomised participants before the specified quit date [15,18,23].

By emphasising that participants should be willing to set a stop date this would prevent randomised participants dropping out of the study before completing the intervention. After having read the information about the study all participants gave informed consent to participate by clicking on a link. After informed consent was obtained the participants were guided to a baseline assessment page. The hyperlink contained within the body of the Email is no longer valid after completion of the questionnaire when the responses are stored in the study database. This prevents multiple responses, while allowing the questionnaire to be completed in more than one session if required. After completion of the baseline assessment the students will be asked to provide their telephone number. Then they will immediately receive a SMS that will have to be responded to by writing, ‘start’, in order to confirm that they had stated the right telephone number.

Participants will immediately be randomised to an intervention or waiting list (control) group. Each participant is allocated a number 1 or 2 with equal probabilities using Java’s built in random number generator (java.util.Random). Randomisation is thus fully computerised, does not employ any strata or blocks, and is not possible to subvert, as this and all subsequent study processes are fully automated.

After randomisation, group 1 will start the intervention immediately with a motivational phase between 1 to 4 weeks, designed to encourage the participants to set a quit date. The participants are given an opportunity to set a quit date every week during this 1 to 4-week motivational phase. If no active quit date is set within this 4-week period, the participant defaults to their originally provided commitment and is given a quit date immediately after the 4-week motivational phase is completed. This procedure is in accordance with the inclusion criteria and informed consent. After setting a quit date the core intervention programme runs for 12 weeks.

Participants in the control group will receive a SMS informing them that they have been allocated to the control group and that they will receive access to the intervention after 4 months. In addition both groups will receive a SMS every fortnight thanking them for participating in the study. Towards the end of the 4-month period both groups are informed that they will receive the follow-up questionnaire in 2 weeks time.

### Blinding

Both groups will be aware that they are participating in a research study and that they will be randomised to an intervention or control group after having confirmed their telephone number. After the 4-month follow-up, the waiting list group will receive access to the intervention. As all study procedures are automated, the research team will have no direct contact with the study participants and so there is no possibility to bias the data collection. The study was approved by the Regional Ethical Committee in Linköping, Sweden (Dnr 2014/217-31).

### Sample size

Based upon previous studies, we expected an absolute difference in cessation rates between the intervention and control group of 5% (with 10% quitting in the intervention group and 5% in the control group) [15,20].

To achieve 80% power with a significance level of 0.05 (2-sided) and correction for continuity, a sample size of 474 persons is needed in each group. If there is 30% attrition in the follow-up measurement the number needed in each group is 677, with the total required sample size being 1,354. The allowance for attrition is deliberately conservative.

### Intervention design and development

Existing guidance for the development of smoking cessation interventions suggests that intervention development should be informed by theory and the existing evidence base [28]. The theoretical models used in previous smoking cessation interventions have mostly been based on social cognitive theory, with tailoring messages by using the transtheoretical model, along with other means of motivational enhancement and psychological support. Supporting self-regulatory skills during the action stage has been shown to be important for a sustainable change of behaviour in young people [9]. However, it remains unclear which particular theories result in the most effective interventions [13], and there is no clear evidence which components or techniques in previous smoking cessation interventions are the most essential to include [29-31].

In the absence of a clear theory to build the intervention, we decided to develop the messages based on existing practice in the form of components of official manuals about smoking cessation recommended in Sweden, as well as using material from books from experienced cessation experts, and key elements from previous SMS-based interventions and Internet- based interventions [12,15,18,20,22-26,32,33]. In order to optimise the participants’ experience of the intervention as a ‘real person’ we were guided by theories of therapeutic relationships [4] and research emphasising the importance of the quality of the actual encounter between client/recipient and professional/caregiver [34,35], as well as on models of the relationships between social interactions, empowerment and health [36]. Efforts were devoted to developing messages that inspired and encouraged participants to increase their ability to mobilise and develop their own resources.

The elements derived from these studies, reviews and interventions used in the present intervention are as follows: making a public declaration about quitting (that is telling friends about the quit attempt); encouraging asking friends and relatives for support; problem solving tips; distraction techniques and the possibility to text for more help if having a slip and starting to crave. All messages were discussed by a multidisciplinary team of researchers with wide experience of life style interventions, in particular SMS- and Internet-based cessation interventions regarding smoking and alcohol.

The basic structure and delivery of the present smoking cessation intervention, called NEXit, was inspired by previous smoking cessation interventions delivered via mobile phone text messaging [17,18,21-25,27]. The central component of these SMS-based interventions was one or more daily text messages tailored to the various phases of quitting smoking: preparation before quitting, the initial quitting phase and the maintenance phase. The length of NEXit was set to 12 weeks in accordance with most previous interventions and recommendations in guidelines [17,18,21-25,27]. In addition to the 12-week core intervention we added a 1- to 4-week motivational phase before setting a stop date, as used in most previous interventions [22-25].

The final NEXit content includes a 1- to 4-week motivational phase and a 12-week core intervention. In the motivational phase the participants receive 2 SMS per day. The messages in the motivational phase contain information relevant in advance of quitting, that is symptoms to expect on quitting, tips to avoid weight gain, tips to cope with cravings and to avoid smoking triggers, motivational support, and how to distract one’s mind from smoking.

The 12-week core programme consists of 157 messages. The participants receive 5 SMS per day 3 days before the quit date. Messages provide information about the consequences of smoking, how to quit and stay having quit. They prompt participants to prepare by getting rid of cigarettes, ashtrays and lighters and to avoid environments where they could normally smoke.

After the quit date the participants will receive 5 SMS per day for the first 3 days and then 4 SMS the rest of the first week. During weeks 2 to 4 the participants receive 2 SMS per day. In weeks 5 to 7 the number of SMS is decreased to 2 SMS every second day and 1 SMS the other days. In weeks 8 to 12 the number of SMS is 1 daily.

Messages encourage participants to persevere with the quit attempt and to focus on their success so far. They are given coping messages for handling cravings, motivational messages, and assistance with withdrawal symptoms. The content covers information relevant to quitting; tips to avoid weight gain and improve nutrition; tips to cope with craving; advice on avoiding smoking triggers; instructions on breathing exercises to perform instead of smoking; motivational support (for example feedback on amount of money and life years saved) and distraction (for example sports, travel, cinema, drinking water).

The intervention includes a function where participants can ask for extra SMS when having problems with craving, relapse or weight gain. By texting the word ‘crave’ participants with cigarette cravings receive three instant messages some minutes apart to distract or support them during these episodes. By texting the word ‘lapse’, participants receive three text messages that encourage them to continue with their quit attempt. By texting ‘weight’, participants receive tips on how to avoid weight gain.

### Earlier work on the feasibility of the intervention

Several steps were taken during the development of the NEXit programme and will be further described here. A first version of the NEXit intervention was developed during the spring of 2013. An expert panel consisting of 10 smoking cessation experts in Sweden evaluated this first version of the intervention during the autumn of 2013 by reviewing all text messages and, based on their feedback, the messages were revised in several iterative steps. Further, a focus group interview with current smokers or smokers who had recently quit was undertaken. The participants were presented with the structure and examples of the content of the programme as part of the interview. The focus group interview was conducted using a semi-structured interview guide in order to collect experiences and points of view regarding structure of the programme, the length, number and content of the messages. The focus group provided generally positive feedback on the structure and content of the intervention.

In the next phase, in late autumn of 2013, 10 smokers were asked to give their opinion about the structure and content of the intervention by reviewing all text messages and giving written feedback to the research team. The purpose of involving smokers individually and in the focus group previously was, in particular, to test and confirm the acceptability of the messages’ number, tone, content and programme duration. Some changes were made to a minority of the messages that were not found appropriate by the smokers. Subsequently, another 8 smokers agreed to test all steps in the intervention from the initial sign-up procedure to the 1- 4-week motivational phase, setting a quit date and receiving the first 4 weeks of the core intervention. Minor revisions were again made.

Based on the above mentioned development steps a second shortened (8 weeks) version of the NEXit intervention was tested in a pilot trial in the winter/spring of 2014 (n = 35) where, besides the content of the messages, the technological feasibility of intervention delivery and study procedures were tested. In the pilot trial all parts of the intervention and data collection were tested and found to work as intended except from some minor but important technical issues that were resolved immediately after the pilot study. After the end of the 8-week intervention, 8 phone interviews were conducted to elicit reactions and opinions on improvements in intervention elements. An interview guide using open-ended questions was used regarding the process of getting started, preparation leading up to quit day, and setting a stop date, as well as on the number of messages, the tone, the timing and the content. The results confirmed that a majority of the participants experienced that the intervention was easy to access, the message content and tone was motivating and helpful, and the intervention included a sufficient number of messages.

Participation in the pilot trial was somewhat lower than expected, and the drop-out at various steps during recruitment and after randomisation was higher than anticipated. Therefore, a number of revisions were made to boost recruitment and prevent attrition before setting a quit date. It was decided that recruitment should not only to be by invitation by mail, but also through posters, leaflets and information on the colleges’ and universities’ homepages during a 1-month period.

### Outcome evaluation

The baseline questionnaire contains 19 questions about sociodemographic data and smoking habits. The participants will be asked to state their age, gender, marital status and years of smoking and, if using Swedish snuff (‘*snus*’), how much per day. Then follows the 6 questions of Fagerström’s Nicotine Dependence Scale and a question on how important it is for the participants to quit smoking with response option on a scale from 1 to 10, where 1 stands for not important at all and 10 very important. All participants will be asked if they ever have tried to stop smoking before (and if so how many times); if they ever have tried nicotine replacement (if so how many times); if they have been prescribed drugs for smoking cessation, and if they have received professional counselling or if they have called the national quit smoking help-line.

After 4 months, a link to an electronic follow-up questionnaire will be Emailed to all participants. Initially, 2 reminders 1 week apart will be sent to non-responders. In order to minimise attrition those participants still not responding will receive additional Email reminders every second day for 6 days (that is 3 Emails). If still not responding, the non-responders will receive a SMS every second day for 6 days (that is 3 SMS) with only 2 questions to answer, to capture the 2 primary outcome measures. Lastly, those still not responding will be phoned with a maximum of 10 calls per participant. Also, in the invitation to answer the follow-up questionnaire the participants will be told that they are offered the opportunity to be part of a draw for a number of iPads (Apple Inc., Cupertino, CA, USA) after having answered the follow-up questionnaire.

Outcome measures are as follows:

*Primary outcome measures*:

1. Self-reported prolonged abstinence (defined as having not smoked more than 5 cigarettes in the last 8 weeks).
2. Self-reported 4-week point prevalence of smoking abstinence (not having smoked a single cigarette).

*Secondary outcome measures*:

1. Self-reported 7-day point prevalence of smoking abstinence (defined as not smoking any cigarettes in the past 7 days).
2. Mean number of quit attempts since taking part in the study.
3. Number of uses of other smoking cessation services (prescribed medication, nicotine replacement medication, counselling, calling the help-line or any other help) since first invitation to the study.
4. Numbers of cigarettes smoked weekly (for participants still smoking at the time of follow-up only.

### Data analysis

The data analysis will conform to the pre-specified statistical analysis plan described below. The analysis will start after collection of the follow-up data. There will be no interim analyses or stopping rules. Following the intention-to-treat analysis strategy, all analyses will include all participants with follow-up data in their groups as randomised, and sensitivity analyses will include all randomised participants to explore different assumptions about the missing data [37].

#### Descriptive analysis

A flowchart of recruitment of participants is displayed in Figure 1. The number of screened students who fulfil the study inclusion criteria, and the number included in the primary and secondary analyses as well the reason for exclusion from these analyses, will be reported. Summary tables (descriptive statistics and/or frequency tables) will be provided for baseline and follow-up variables, as appropriate. Continuous variables will be summarised with descriptive statistics (n, mean, standard deviation for data with normal distribution, or median and interquartile range for non-normally distributed data). Frequency counts and percentages of subjects within each category will be provided for categorical data. Visual inspection of box plots of number of quit attempts and number of cigarettes smoked daily (if still smokers) will be used to identify possible outliers to be excluded in sensitivity analyses.

#### Primary analyses

The primary analyses will assume that missing outcome data are missing at random and, therefore, will be performed on complete cases only.

The binary outcomes of self-reported prolonged abstinence, 4-week prevalence of smoking abstinence and 7-day point prevalence of smoking abstinence will be analysed by logistic regression and results presented as odds ratio (95% CI). Number of quit attempts and number of uses of other smoking cessation services will be analysed by negative binomial regression and results presented as ratio of means (95% CI). Numbers of cigarettes smoked weekly will be analysed by logarithmic transformation and linear regression and results presented as ratio of geometric means (95% CI).

All regression analyses will be adjusted for by the following baseline variables: gender, years of smoking, average number of cigarettes smoked weekly, severity of dependence as measured by Fagerström’s Nicotine Dependence Scale and amount of ‘*snus*’ used at baseline.

Effect modification analyses will be performed for the two primary outcomes and the following potential effect modifiers measured at baseline: gender, average number of cigarettes smoked weekly, amount of ‘*snus*’ used weekly, and severity of dependence as measured by Fagerström’s Nicotine Dependence Scale. Each effect modification will be assessed by adding the appropriate interaction term to the adjusted regression model. Consideration for adjustment for multiplicity of comparisons will be discussed. All tests will be performed 2-sided with a 5% level of significance.

#### Sensitivity analyses

Sensitivity analysis will explore the effects of departures from the missing at random assumption in the main analysis [37-40]. As suggested by Jackson *et al*. [39], we will quantify departures from the missing at random (MAR) assumption by the informatively missing odds ratio (IMOR). We will assume that the IMOR is the same in each randomised group and we will vary the IMOR over the range 0.5 to 1; if 10% of observed data are abstinent then this range implies that from 5% to 10% of missing data are abstinent. We will also allow the IMOR to be 0 (missing = smoking, the Russell standard) [41]. Further, we will use data on the number of follow-up Emails, texts and phone calls needed before an individual responded to explore the plausibility of the MAR assumption: first by exploring the association between quitting and number of follow-up attempts needed, and then by fitting the repeated attempts model of Jackson *et al*. [38,39] which will allow us to both estimate the degree of departure from MAR and to adjust for departure from MAR.

In associated methodological work, we will also use the data on the number of follow-up Emails, texts and phone calls needed before an individual responded to explore: 1) the implications of a less intensive data collection regime, and 2) the validity of the assumptions of the repeated attempts model, including that data collected at later attempts are of equal quality.

## Discussion

All colleges and universities in Sweden have a student health care centre with a responsibility to undertake preventive work with regards to alcohol, tobacco and other drugs. Our research group has previously performed a number of research and development studies concerning alcohol Internet-based interventions with the student health care centres in all parts of Sweden [42-46]. There is a general absence of smoking cessation interventions specifically targeting young people in Sweden and the student health care centres have limited resources for individual support to smokers, and thus have not been able to focus sufficiently on helping smokers who want to quit. Thus, there is a need for this study in Sweden, and the data from this study will also contribute to the international literature.

As well as providing substantive data on intervention effectiveness, this study will also provide data for methodological analyses addressing a number of issues commonly challenging in Internet-based RCTs. We will explore whether outcomes differ depending upon when an individual responds to follow-up and if data collected after repeated attempts are of a lower quality. Also, we will explore how an exhaustive approach to follow-up, with multiple frequent reminders, influences the extent of missing data and its handling. Furthermore, using the methods of Jackson *et al*. [47] we will explore whether the Russell standard [41], assuming missing outcome data to mean still smoking, is reasonable or whether a MAR assumption is more appropriate in these data. This will give new insights on how to treat missing data in RCTs of smoking cessation interventions.

One limitation of the study is that smoking status will be assessed by self-report and not be biochemically verified. Over-reporting of smoking abstinence may be assumed to be equal in both the intervention and control group. The Society for Research on Nicotine and Tobacco (SRNT) suggest that in population-based studies with limited face-to-face contact, it is neither required nor desirable to use biochemical verification [48].

## Trial status

At the time of submission the recruitment of participants had started but is not yet complete. Recruitment will be completed on the 14 November 2014.

## Additional file

Additional file 1:
**SPIRIT 2013 Checklist explaining where recommended items are addressed in the trial protocol.**

## Abbreviations

CI: Confidence interval
CONSORT: Consolidated Standards of Reporting Trials
IMOR: Informatively missing odds ratio
MAR: Missing at random
NEXit: Nicotine EXit - the name of the intervention
RCT: Randomised controlled trial
SMS: Short message service - more popularly called text messages
SRNT: The Society for Research on Nicotine and Tobacco
